# Problematic Alcohol Use and Mortality Risk in Arrhythmia: Nationwide Study of 114,958 Hospitalizations

**DOI:** 10.7759/cureus.8194

**Published:** 2020-05-19

**Authors:** Khalida Khaliq, Temitope Ajibawo, Renu Bhandari, Rikinkumar S Patel

**Affiliations:** 1 Psychiatry/Medicine, North Tampa Behavioral Health, Tampa, USA; 2 Internal Medicine, Brookdale University Hospital Medical Center, New York City, USA; 3 Medicine, Manipal College of Medical Sciences, Kaski, NPL; 4 Psychiatry, Griffin Memorial Hospital, Norman, USA

**Keywords:** alcohol misuse, alcohol dependence, alcohol use, mortality, cardiac risk factors and prevention, life-threatening arrhythmia

## Abstract

Objectives

To assess the risk of in-hospital mortality due to alcohol use disorder (AUD) and other cardiovascular risk factors in arrhythmia inpatients.

Methods

We included 114,958 patients (age, 15-54 years) by conducting a cross-sectional cohort study using the Nationwide Inpatient Sample (NIS, 2010-2014). These patients were primarily managed for arrhythmia and further grouped by comorbid AUD. A logistic regression model was used to measure the odds ratio (OR) of association of AUD and in-hospital mortality after adjusting for demographic confounders and cardiovascular risk factors.

Results

Mortality risk statistically increases with age as elders (45-54 years) had two times higher risk (95% confidence interval [CI] 1.49-3.09), whereas men had a lower risk (OR 0.8, 95% CI 0.74-0.96) of inpatient death. Comorbid atherosclerosis (OR 4.5, 95% CI 3.38-5.92) and diabetes (OR 1.4, 95% CI 1.18-1.67) increased mortality risk in arrhythmia inpatients. AUD significantly increased the risk of mortality in arrhythmia inpatients (OR 1.7, 95% CI 1.43-2.07).

Conclusions

AUD is an independent risk factor for mortality in arrhythmia inpatients, and it is elevated by 72% in such patients. Strategies to reduce alcohol consumption and abstinence should be focused to improve the health-related quality of life of at-risk patients.

## Introduction

Arrhythmia is a frequently seen cardiac condition in clinical settings. The heart rhythm can be erratic, or too slow known as bradycardia, or too fast known as tachycardia. Supraventricular arrhythmias are seen in 35 cases per 100,000 patients with a prevalence of 2.25% per 1,000 in the general population [1]. On the other side, atrial fibrillation (AFib) is commonly seen as arrhythmias in inpatient settings and the emergency department (ED), affecting two million Americans. Ventricular arrhythmias are most lethal, leading to sudden cardiac death in 80% of the cases. The common causes of arrhythmias are coronary artery disease, hypertension electrolytes imbalance, injury to the heart, alcohol consumption, and substance abuse [1]. There are two different essential disturbances found in the pathophysiology of arrhythmias: changes in automaticity (impulse formation) and impulse conduction or both [2].

Alcohol abuse and dependence are some of the serious burdens in the United States (US) healthcare system [2]. After tobacco abuse, alcohol abuse is the second frequent form of substance abuse and is accountable for approximately 88,000 deaths annually. An estimated 14.5 million, i.e. 5.8% of the adult population (9.2 million men and 5.3 million women) abuse alcohol [3,4]. Excessive alcohol consumption adversely affects the cardiovascular system, leading to hypertension, alcoholic cardiomyopathy, stroke, and arrhythmias. The most frequent forms of arrhythmia seen in alcohol abusers are supraventricular arrhythmia and AFib, and occasionally ventricular arrhythmia in chronic alcoholics [3].

There are a few underlying mechanisms involved in the development of arrhythmias due to excessive alcohol consumption. Ethanol and its metabolite have a profound effect on the heart due to excessive formation of free radicals, increased oxidative stress and protein breakdown, and apoptosis of the cells [5]. These lead to myocardial damage alcoholic cardiomyopathy and delay in cardiac conduction by facilitating the re-entry phenomena. The localized area of myocardial damage results in a patchy distribution of the lesion and patchy conduction delay in cardiac impulse conduction. Also, increased adrenergic activity is seen in alcohol abusers, which increases the heart rate causing arrhythmias [5,6].

Arrhythmias are accountable for an estimated 200,000 to 300,000 sudden cardiac deaths annually in the US [7]. Alcohol use disorder (AUD), including abuse and/or dependence, is one of the important causes of morbidity and mortality associated with cardiac arrhythmias [8,9]. Few studies have shown that excessive chronic alcohol consumption, as well as acute alcohol abuse, is associated with increased risk of arrhythmias [7-9].

No studies in the past have explored the risk of AUD as an independent predictor for in-hospital mortality in arrhythmia. In our study, we aim to evaluate the differences in demographics and comorbidities seen in arrhythmia inpatients by comorbid AUD and to assess the risk of in-hospital mortality due to AUD and other cardiovascular comorbidities in arrhythmia inpatients.

## Materials and methods

Data source

We used the Nationwide Inpatient Sample (NIS) data from 2010 to 2014 in our cross-sectional analysis. The NIS provides clinical information from about 4,400 community hospitals across 44 states in the US, and the diagnostic information is based on the International Classification of Diseases, Ninth Edition (ICD-9) codes, and Clinical Classification Software (CCS) codes [10].

Inclusion criteria and outcome variables

We included 114,958 patients (age 15-54 years) with a primary discharge diagnosis of arrhythmia using ICD-9 codes 427.0-427.2, 427.31, 427.32, 427.60, 427.61, 427.69, 427.81, 427.89, 427.9, 785.0, or 785.1. The study inpatients were further grouped by comorbid discharge diagnosis of AUD (including alcohol abuse or alcohol dependence) using the ICD-9 codes 291.0-291.3, 291.5, 291.8, 291.81, 281.82, 291.89, 291.9, 303.00-303.93, or 305.00-305.03.

Demographic variables studied included age (15-34, 35-44, and 45-54 years), sex (male and female), and race (white, black, Hispanic, and others) [11]. Based on current literature, we selected comorbid diagnosis of diabetes, hypertension, obesity, elevated cholesterol and lipids, and tobacco abuse that were identified using ICD-9 or CCS diagnosis codes [11,12]. We measured all-cause in-hospital mortality between AUD and non-AUD cohorts [11].

Statistical analysis

We used descriptive statistics and Pearson’s chi-square test to measure the demographic and comorbidities differences in arrhythmia inpatients by comorbid alcohol abuse. Logistic regression analysis was used to evaluate the demographic and cardiovascular comorbidities and comorbid AUD that increase the risk of association with in-hospital mortality. A P-value of less than 0.01 was used to determine the statistical significance, and data analysis was done using Statistical Package for the Social Sciences (SPSS) version 26 (IBM Corporation, Armonk, NY).

Ethical approval

To protect the patient's identity and health information, the NIS used separate codes to de-identify the database [10]. We do not require approval from the institutional review board.

## Results

We analyzed a total sample of 114,958 inpatients hospitalized for arrhythmia with 9.75% having comorbid AUD. The majority of the inpatients with AUD were older adults aged 45 to 54 years (68%), and were older than non-AUD (45.8 years vs. 43.9 years, P < 0.001). A higher proportion of arrhythmia inpatients with comorbid AUD were males (85.9% vs. 61.5%) and white (69% vs. 65.8%) as compared to the non-AUD cohort. Comorbidities were seen in a lower proportion of AUD cohort other than a statistically significant greater difference with hypertension (52.2% AUD versus 46.4% non-AUD) and tobacco abuse (42.3% in AUD vs. 17.4% in non-AUD) (Table 1).

**Table 1:** Demographic and comorbidities distribution, and in-hospital mortality in arrhythmia inpatients N: number of patients, AUD: alcohol use disorder, SD: standard deviation

Variable	AUD (no)	AUD (yes)	Total	P-value
Total N	103744	11214	114958	-
Mean age, years (SD)	43.9 (9.52)	45.8 (8.37)	-	<0.001
Age at admission, in %				
15–24 years	5.9	2.6	5.6	<0.001
25–34 years	11.1	10.5	11.1	
35–44 years	22.3	18.9	22.0	
45–54 years	60.6	68.0	61.3	
Sex, in %				
Male	61.5	85.9	63.9	<0.001
Female	38.5	14.1	36.1	
Race, in %				
White	65.8	69.0	66.1	<0.001
Black	18.8	16.3	18.6	
Hispanic	9.7	10.2	9.7	
Other	5.7	4.5	5.6	
Comorbidities, in %				
Obesity	23.0	16.1	22.3	<0.001
Diabetes	16.2	11.7	15.7	<0.001
Hypertension	46.4	52.2	47.0	<0.001
Elevated cholesterol and lipids	27.6	22.6	27.1	<0.001
Atherosclerosis	1.5	1.5	1.5	0.969
Tobacco abuse	17.4	42.3	19.9	<0.001
In-hospital mortality, in %	0.9	1.4	0.9	<0.001

The risk of in-hospital mortality statistically increases with increasing age. Older adults (age 45-54 years) have two times higher odds (95% confidence interval [CI] 1.49-3.09) compared to young inpatients (age 15-24 years). Males are at lower risk compared to females (odds ratio [OR] 0.84, 95% CI 0.74-0.96). Blacks are at 1.1 times higher odds as compared to whites (95% CI 1.30-1.77); in contrast, a statistically non-significant association exists between Hispanic and mortality risk (P = 0.118). Comorbid atherosclerosis (OR 4.48, 95% CI 3.38-5.92) and diabetes (OR 1.41, 95% CI 1.18-1.67) were significantly associated with mortality risk.

In-hospital mortality was seen in a significantly higher proportion of the AUD cohort compared to non-AUD (1.4% vs. 1.9%, P < 0.001). AUD significantly increases the risk of in-hospital mortality (OR 1.72, 95% CI 1.43-2.07) compared to the non-AUD cohort after controlling for demographic confounders and cardiovascular risk factors (Table 2).

**Table 2:** In-hospital mortality risk in arrhythmia inpatients

Variable	Logistic regression model			
	Odds ratio	95% confidence interval		P-value
		Lower	Upper	
Age at admission				
15–24 years	Reference			
25–34 years	1.64	1.09	2.44	0.016
35–44 years	1.69	1.16	2.48	0.006
45–54 years	2.15	1.49	3.09	<0.001
Sex				
Male	0.84	0.74	0.96	0.011
Female	Reference			
Race				
White	Reference			
Black	1.52	1.30	1.77	<0.001
Hispanic	1.19	0.96	1.48	0.118
Other	1.59	1.25	2.03	<0.001
Alcohol use disorder				
No	Reference			
Yes	1.72	1.43	2.07	<0.001
Comorbidities				
No comorbidity	Reference			
Obesity	0.82	0.69	0.97	0.020
Diabetes	1.41	1.18	1.67	<0.001
Hypertension	0.91	0.79	1.05	0.193
Elevated cholesterol and lipids tobacco abuse	0.48	0.40	0.57	<0.001
Atherosclerosis	4.48	3.38	5.92	<0.001
Tobacco abuse	0.94	0.80	1.11	0.481

## Discussion

Arrhythmia inpatients with AUD were prevalent in elderly white males. Atherosclerosis and diabetes significantly increase mortality risk in arrhythmia inpatients. Mortality was seen in a higher proportion of alcohol abusers (1.9%) compared to non-users (1.4%) and AUD increased the risk of in-hospital mortality by 72% due to the arrhythmogenic effect of excessive alcohol consumption (acute or chronic) on the heart.

Approximately 9.75% of inpatient hospitalization for arrhythmias had comorbid AUD. Past studies found a strong association between the AUD and arrhythmia with an incidence rate of 17.3% and 20.8% per 1,000 persons-years with moderate and heavy alcohol consumption, respectively. There is a significant increase of 8% relative risk of incidence of arrhythmia for each drink per day as compared to non-alcoholics [13,14]. A higher proportion of the arrhythmia inpatients with comorbid AUD were older males aged between 45 and 54 years (68%) as the risk of arrhythmia is three times higher in men and middle aged/elderly people [15]. Men are at two times higher risk for alcohol abuse than women, and the prevalence increases with age; this was reflected in our study as 85.9% of males with arrhythmia had comorbid AUD [15].

The incidence and prevalence of arrhythmias subtypes differ by gender, as AFib and Wolff-Parkinson-White (WPW) syndrome are more frequently seen in males, while atrioventricular nodal tachycardia seen more in females. This difference in incidence most likely related to the difference in hormonal effect on ion channels in myocytes during repolarization and difference in cardiac electrophysiology. Although males are more prone to have arrhythmias, with a higher incidence of AFib in males, females are at higher mortality risk compared to males [16,17]. Women are more prone to higher morbidity and mortality at a low level of alcohol consumption than men [18]. This effect is related to lower total body water in females and attaining a high level of blood alcohol concentration, the difference in alcohol metabolism, and hormonal effect of alcohol in postmenopausal women [18].

The risk of arrhythmia varies by races and/or ethnicities. The majority of arrhythmia inpatients with AUD were whites (69%). The lifetime risk of arrhythmias in a white is one in three as compared to one in five in blacks. This difference is most likely due to limited access to healthcare in minorities. It is found that Asians have the lowest odds of high alcohol consumption followed by Latinos, and Hispanics, whereas whites found to have the highest odds of excessive acute and chronic alcohol consumption. In our study, we found that blacks have higher odds of in-hospital mortality during arrhythmia hospitalization. Past studies also found that the risk of mortality in arrhythmias is higher in blacks compared to whites, and variable in Hispanics but lowest in Asians due to limited access to healthcare, high prevalence of comorbidities like hypertension, diabetes, obesity, and substance abuse that may have a synergetic effect on cardiac arrhythmias [19,20].

Many risk factors and chronic comorbidities that involve in the progression of arrhythmias are hypertension, diabetes, obesity, atherosclerosis, alcohol, and tobacco abuse [12]. In our study, we found a significant proportion of hypertension and tobacco abuse in arrhythmia inpatients with AUD compared to the non-AUD cohort. Hypertension is one of the independent major risk factors with the prevalence of 40%-90% in arrhythmia, and as per the Framingham's heart study, the risk of arrhythmias is not only associated with stage II-IV hypertension but also with borderline hypertension. Obesity is another important independent risk factor for arrhythmias, but after controlling for other risk factors we found a statistically non-significant association with mortality. We have found 23% obese arrhythmia in a non-AUD cohort compared to 16.1% in arrhythmia inpatients with AUD. The Framingham heart study illustrates that for each unit increase in the body mass index (BMI) there is a 4% increase in the risk of arrhythmias, yet we did not find a significant association of mortality and obesity [21].

In our study, we found 16.2% of diabetics in non-AUD compared to 11.7% in the AUD cohort were hospitalized for arrhythmia. The underlying mechanism of the high risk of mortality is complex, and it is a combination of autonomic dysfunction secondary to diabetic neuropathy, fibrosis, and fat deposition left ventricular hypertrophy and remodeling of heart that leads to fatal and non-fatal arrhythmias. Atherosclerosis is another significant risk factor associated with arrhythmia development and progression. In our study, we found that 1.5% AUD and non-AUD arrhythmia inpatients had atherosclerosis, yet these patients had 4.5 times higher risk of in-hospital mortality, possibly due to coronary artery hypoperfusion, atrial remodeling, and ventricular remodeling leading to fatal arrhythmias [22].

AUD is associated with an increased risk of ventricular arrhythmias and sudden cardiac death. Alcoholics have a higher prevalence of comorbidities, including hypertension, obesity, diabetes, atherosclerosis, depression, and substance abuse, compared to non-alcoholics. These comorbidities in patients with AUD have a vicious effect that increases the risk of mortality due to impaired vagal activity, and impaired baroreceptor sensitivity leading to fatal arrhythmias. The electrolyte abnormalities like hypomagnesemia and hypokalemia in alcoholics primarily or secondary to malnutrition, nausea, vomiting, diarrhea, and dehydration result in the prolonged QT interval and torsade de pointes that causes life-threatening arrhythmias [8,9]. It is interesting to see that in our study comorbid tobacco abuse was highly prevalent in arrhythmia inpatients with AUD, and alcohol and tobacco have a synergetic adverse effect on cardiovascular health. The pharmacological and behavioral effects of alcohol and nicotine in tobacco potentiate the rewarding effect along with the cross-tolerance effect via the shred gene and cross reinforcement via the limbic pathway [23]. In our study, we found higher in-hospital mortality in arrhythmia inpatients with AUD (1.9%) compared to the non-AUD cohort (1.4%). After controlling for demographic confounders and chronic comorbidities, AUD is an independent risk factor that increases the risk of in-hospital mortality by 1.7 times as compared to the non-AUD cohort.

Few limitations in our study include the underreporting of AUD, due to inconsistency in administrative data based on ICD-9 codes, including diagnostic codes during patient billing. Also, the variable included to measure in-hospital mortality is all-cause and so our study does not show a causal relationship between mortality and comorbidities in arrhythmia patients. There were few recent studies that found cannabis is an independent cardiovascular risk factor for arrhythmias and myocardial infarction, but we were not able to control our regression model with cannabis abuse or dependence [24-26]. Some of the major strengths our study include nationwide data analysis covering 44 states across the US; moreover, our results have appropriate external validity to the American population and a strong methodology including a demographic adjusted regression model to evaluate the risk of association with mortality.

## Conclusions

Alcohol is one of the major factors for the global burden of the disease with deleterious effects on physical health with higher morbidity and mortality. The risk of mortality is higher in older adults and females with arrhythmia. AUD independently increases the risk of mortality by 72% in arrhythmia inpatients. Social alcohol drinking and its impact on arrhythmias in non-alcoholics need to be studied. Strategies to reduce alcohol consumption and abstinence should be focused to improve the health-related quality of life of patients with cardiovascular risk factors. Education must be provided to patients with alcohol use problems regarding the adverse effects of AUD on physical and mental health along with the appropriate resources and support.
